# Development of an Online-Coaching Blended Couple-Oriented Intervention for Preventing Depression in Middle Adulthood: An Intervention Mapping Study

**DOI:** 10.3389/fpubh.2022.882576

**Published:** 2022-06-02

**Authors:** Suk-Sun Kim, Minji Gil, Daeun Kim

**Affiliations:** ^1^Ewha Research Institute of Nursing Science, College of Nursing, Ewha Womans University, Seoul, South Korea; ^2^College of Nursing, Ewha Womans University, Seoul, South Korea

**Keywords:** intervention mapping, program development, online intervention, couples therapy, primary prevention, depression, middle aged adult

## Abstract

**Background:**

Although middle-aged adults in Korea are vulnerable to depression, there are few preventive interventions for depression in middle adulthood. Studies consistently suggest that interventions that include both spouses are effective in decreasing depression and relationship distress. Considering the busy lives of middle-aged couples, it is essential to develop an online-coaching blended couple-oriented intervention. This study aimed to describe the development, implementation, and evaluation of an online-coaching blended couple-oriented intervention using an online program and coaching *via* videoconference to prevent middle-aged couples' depression; this was done using an intervention mapping (IM) protocol.

**Methods:**

Six steps of IM were used to systematically develop a tailored multi-level intervention specific to middle-aged couples' depression. These steps of the IM protocol involve needs assessment, formulation of change objectives, theory-based methods, and practical strategies for program design, program development, program implementation, and program evaluation.

**Results:**

The results of the six steps were as follows: (a) middle-aged couples' needs and mental health problems were identified through a scoping review study, mixed-method study, and expert interviews; (b) six performance objectives (POs) were formulated based on the results of Step 1, and intrapersonal, interpersonal, and temporal/transpersonal determinants were identified based on the self-transcendence theory. Change objectives were developed by combining POs with determinants; (c) self-regulated learning was chosen for theoretical teaching methods and practical strategies to change the determinants of each level; (d) four modules consisting of 16 sessions were developed based on the self-transcendence theory; (e) experts evaluated the program and coaches were trained; and (f) the evaluation plan for the program's feasibility, acceptability, usability, and preliminary effects was developed.

**Discussion:**

The systematic process using IM allowed us to develop an online-coaching blended couple-oriented intervention to prevent depression and promote couples' relationships. The primary effects of this newly developed program should be evaluated in future studies. This may lead to the increased adoption and implementation of evidence-based and tailored interventions for psychological wellbeing in middle adulthood.

## Introduction

Depression is a common mental disorder that contributes to the overall global disease burden (1, 2). There is growing concern about the increasing prevalence of depression among middle-aged Koreans (3). The prevalence of depression in middle-aged adults has gradually increased from ~220,000 people in 2016 to 250,000 people in 2020 (4).

Many studies have identified the risk factors for middle-aged depression, including retirement, economic insecurity, changes in family structure, empty-nest syndrome, declining health, hormonal changes, sexual dysfunction, loneliness, and loss of social capital (5, 6). Previous research suggests that chronic exposure to risk factors makes middle-aged adults more vulnerable to depression (6). Because middle-aged adults in Korea often remain undetected and untreated despite their vulnerability to depression (3), it is important that preventive interventions for depression are tailored for a particular target group.

Couple-oriented interventions, targeting couple relationships, involved both spouses of committed relationships in conjoint sessions (7). Compared with individual-oriented interventions for depression focusing on individual cognitive and behavioral changes, couple-oriented interventions appeared more effective in improving depressive symptoms and enhancing couple relationships regardless of the degree of distress (7).

Common couple-oriented interventions for depression include traditional behavioral couple therapy (8), cognitive-behavioral couple therapy (9, 10), and emotionally focused couple therapy (11). However, recent research has emphasized that the integration of cognitive-behavioral couple therapy with communication, problem-solving training, and emotional acceptance is more effective in reducing depression and improving couple relationships (12, 13). Moreover, the development of integrated couple-oriented interventions is required to reflect the mental health needs of vulnerable populations across life stages, particularly in middle adulthood.

Furthermore, to increase the reach of couple-oriented interventions, researchers have developed blended interventions that combine both online self-help approaches, such as digital video lessons and practical materials, and online coaching approaches, such as phone calls and videoconferences with psychologists (12, 13). Blended interventions can reach more couples than traditional therapy due to reduced financial costs, time, and geographic restrictions (13, 14). Considering the busy lives of middle-aged couples and the COVID-19 pandemic era (15, 16), it is vital to develop a blended couple-oriented intervention adapted to an online self-help program with coach support to prevent depression and promote couple relationships.

This study describes the development of an online-coaching blended couple-oriented intervention using an online program and coaching for middle-aged couples' depression by applying intervention mapping (IM). The systematic planning protocol of IM has been widely used by many researchers to develop mental health programs (17), such as art therapy programs for patients with personality disorders (18), media smoking prevention programs for female high school students (19), and mental health education programs for pharmacy staff (20). However, to date, there have been few interventions applying the IM protocol to develop tailored interventions for middle-aged couples' depression. Therefore, the purpose of this study was to describe the development, implementation, and evaluation of an online-coaching blended couple-oriented intervention using an online program and coaching to prevent depression in middle-aged couples.

## Methods

Intervention mapping provides guidelines for systematically developing, implementing, and evaluating new programs based on a foundation of theory, empirical evidence, and the needs of the target population (16). The process of program planning of IM includes six steps: (a) identifying needs or health problems by conducting needs assessment; (b) creating a matrix of change objectives by combining performance objectives with determinants; (c) choosing theoretical methods and translating these into practical strategies or applications; (d) generating a program including scope and sequence of the components of the intervention and preparing program materials and protocols; (e) planning the adoption and implementation of the program; and (f) planning the evaluation of the newly developed program and its implementation.

This study focuses mainly on the results of steps 1–4 regarding the development of a new online-coaching blended couple-oriented intervention using an online program and coaching *via* videoconference, called the *MindGuide Couple* program. This study was approved by the Institutional Review Board of Ewha Womans University.

## Results

### Step 1. Needs Assessment

To identify middle-aged couples' needs regarding the prevention of depression and promotion of psychological wellbeing, we conducted a needs assessment by integrating a scoping review, mixed-method study, and expert interviews.

#### Scoping Review

We systematically reviewed the characteristics, content, theory, and outcomes of couple-oriented interventions for both mental health promotion and the primary prevention of mental disorders. The protocol for this scoping review has been previously published (21). The results of the scoping review showed that couple-oriented interventions were classified as cognitive-focused (e.g., cognitive existential couple therapy), behavior-focused (e.g., integrative behavioral couple therapy), emotional-focused (e.g., emotionally focused couple therapy), communication-focused (e.g., couple-based supportive communication), and relationship-focused (e.g., couple and relationship education). These interventions targeted cancer patients and their spouses, couples with a specific problem (e.g., chronic pain, HIV, alcohol problems, relationship distress, and low income), and couples over the age of 18 years. However, interventions targeting specific age groups (e.g., young, middle-aged, and older couples) have not been found. Considering the high risk of middle-aged depression in South Korea, couple-oriented interventions to prevent depression in middle adulthood need to be developed.

Moreover, most interventions were conducted in the U.S.A. and delivered face-to-face. These findings indicate that there is a need to develop culturally tailored interventions for Asians and transition from face-to-face to online or blended interventions, considering the COVID-19 pandemic.

#### Need Assessment of Middle-Aged Couples

To determine and prioritize educational needs to promote middle-aged couples' psychological wellbeing, an exploratory sequential mixed-method study was conducted.

First, a qualitative study was conducted through couple interviews with 14 middle-aged couples who were recruited using the snowball sampling method. Open-ended questions regarding the experience of psychological wellbeing were asked, such as “What psychological wellbeing do you experience in middle adulthood?” and “Could you describe what is needed to improve your or your spouse's psychological wellbeing?” Three major themes were identified: (a) confronting vulnerability to mental health problems; (b) navigating life's journey together; and (c) progressing toward self-transcendence. Middle-aged couples were aware of the importance of mental health in middle adulthood while experiencing a midlife crisis as they aged. They overcame the midlife crisis by forming a We-ness in which couples have a depth of connection and support each other. Moreover, they moved toward self-transcendence based on the expansion of relationships with the self, others, environments, and a higher being.

Second, a self-administered questionnaire was developed based on the qualitative results and a literature review. The questionnaire included six competencies of psychological wellbeing: mental health, emotional regulation, spirituality, couple communication, and couple relationships, as well as barriers and facilitators of the couple-oriented intervention. Overall, 234 middle-aged adults were recruited *via* advertisements on several social media platforms. The inclusion criteria were as follows: (a) 40–65 years of age; and (b) individuals in a committed relationship. The Borich Needs Assessment Model compared differences between participants' perceptions of the importance of competencies regarding psychological wellbeing and their ability to perform these competencies. The priority needs found as follows: (a) couple communication issues (i.e., continuous communication, speaking, and listening); (b) mental health issues (i.e., stress, depression, anxiety, and suicide); (c) emotional issues (recognizing accepting and expressing emotions); (d) couple relationship issues (i.e., conflict, understanding the spouse, and leisure with the spouse); (f) change in middle age issues (i.e., role change, physical and psychological change); and (e) spirituality issues (i.e., meaning in life, altruistic life, and self-integration). In addition, 80.3% of the participants answered that couple-oriented intervention was necessary, but 91.9% did not participate because they were too busy and did not have enough time in their schedule.

Finally, the integrated results of the qualitative and quantitative study indicated that middle-aged couples could progress toward self-transcendence by expanding multidimensional boundaries in order to overcome vulnerabilities to depression. In addition, couple-oriented interventions for preventing depression should teach effective communication skills to help participants express emotions and thoughts to their spouses, which leads to the formation of We-ness in couples and promotes psychological wellbeing. The detailed results of this study have been previously reported in Korea (22).

#### Expert Interview

The expert interview was conducted with eight marriage and family therapists, including a psychiatrist, two psychiatric mental health nurses, and five psychologists. They had worked as marriage and family therapists for 8–30 years. Weekly counseling sessions have been reported to range between 1 and 20. Semi-structured interview questions included (a) reasons to start couple therapy; (b) benefits of couple therapy; (c) preferred techniques; and (d) the roles of couple therapists. They engaged in interviews at their workplace or over the phone for a duration of 45–73 min. The qualitative text analysis software MAXQDA was used to code and analyze the data using content analysis.

First, most therapists stated that couples received couple therapy for relationship distress. Couples with distress believe that relationship problems are caused by personal differences. However, therapists noted that weak management of couple conflict might lead to relationship problems, as well as poor mental health issues, such as depression.

Second, therapists said that couple therapy focuses on strengthening couples' relationships by equipping them with communication and problem-solving skills. Couple therapy helped couples to understand each other better and improve attachment and bonding with the spouse, which built more resilient relationships. Relational resilience can play a protective role in promoting mental health.

Third, the experts reported that integrative techniques from different therapies were used depending on clients' needs or problems since integrative techniques provide flexibility in couple therapy and offer the opportunity to improve the efficacy of therapy. They used a family tree, imago conversation, healing inner child, mindfulness, cognitive restructuring, role-playing, and a gratitude diary. Moreover, they highlighted the importance of combining individual and conjoint sessions with one therapist in all sessions. As marriage problems are prone to be interactive, for one spouse to make a change for the better, both spouses need to work together.

Fourth, therapists emphasized that building a solid therapeutic alliance with a couple is a critical component of successful couple therapy. They also said that the primary role of a couple therapist is as a facilitator who helps couples strengthen relationships by understanding the spouse better and focusing on the strengths of a couple. They helped a couple enhance the emotional bond between spouses by caring about each other and navigating and resolving conflicts effectively.

#### Integrated Results

Based on the integrated findings from the scoping review, mixed-method study, and expert interviews, the research team discussed and identified the target population, purpose of the intervention, delivery method, and techniques. First, we found that it is necessary to develop a couple-oriented intervention targeting middle-aged couples' depression because they are at a higher risk of depression (3, 4), and their negative relationship influences depression. Second, the integrated results suggest that increased self-transcendence might promote psychological wellbeing and prevent depression. Hence, the goal of the intervention is to expand self-boundaries multidimensionally, according to the self-transcendence theory (23). Third, considering the busy lives of middle-aged couples, an online-coaching blended couple-oriented interventions using an online program and coaching should be developed to meet their needs. Finally, the findings highlight that integrating techniques in line with Korean culture increased the effectiveness of couple-oriented interventions for depression in couple relationships.

### Step 2. Formulation of Change Objectives

Performance objectives (POs) were formulated to develop a new tailored intervention for preventing mental disorders, such as depression, and enhancing couple satisfaction for middle-aged couples based on the integrated results of step 1. POs are more specific than the traditional goal of a program because their statements include those who need to change and what needs to be changed (17).

A matrix of change objectives (COs) was developed by combining POs with determinants (Table 1). We selected intrapersonal, interpersonal, and temporal/transpersonal determinants derived from the self-transcendence theory (23). To prevent depression and enhance couple satisfaction for middle-aged couples by expanding boundaries, the intervention should increase knowledge, attitudes, and skills at an intrapersonal level, as well as relationship functioning at an interpersonal level and shared outcomes at a temporal/transpersonal level. Finally, we identified 32 COs that indicated the intervention.

**Table 1:** Matrix of change objectives for *MindGuide Couple* Program.

**Performance objectives**	**Intrapersonal determinants**			**Interpersonal determinants**	**Temporal/** **transpersonal determinants**
	**Knowledge**	**Attitude**	**Skill**	**Relationship functioning**	**Shared outcome**
PO1. Understanding mental health issues in middle adulthood	K1a. Understanding vulnerability to depression in middle adulthood K1b. Describing depressive symptoms K1c. Learning about the key suicide signs and warnings	A1a. Having positive attitude toward program A1b. Having empathy for people with depression or mental disorder	S1a. Performing a screening for depression	R1a. Recognizing depression in spouse	
PO2. Trying coping strategies for preventing depression	K2a. Knowing about healthy coping techniques	A2a. Getting motivated through the perceived benefits of healthy coping techniques	S2a. Developing healthy coping techniques		
PO3. Becoming more self-aware	K3a. Being aware of emotion, thought, and behavior	A3a. Cultivating a positive perspective in life through reflecting on the past	S3a. Managing the emotion, thought, and behavior effectively	R3a. Recognizing you and your partner's differences	
PO4. Building a healthy communication in couple relationships	K4a. Understanding positive and negative communication style	A4a. Communicating respectfully with your spouse	S4a. Sharing your needs and feelings in a respectful way	R4a. Understanding more deeply your spouse R4b. Becoming aware of your own communication style as well as your partner's communication style. R4c. Building empathy and improving communication skill	SO4a. Working together to resolve relationship conflicts SO4b. Respecting the needs of both partners
PO5. Making the therapeutic alliance in couple				R5a. Helping your spouse heal their emotional wounds R5b. Showing respect to your partner R5c. Providing emotional support R5d. Remembering you love each other	SO5a. Facilitating healing in a transpersonal connection SO5b. Supporting each other to grow together
PO6. Rebuilding life's meaning	K6a. Understanding your personal values and life meaning	A6a. Being strong and moving forward with a positive attitude		R6a. Making blueprint together as a couple	SO6a. Understanding both spouses' values and their role in life SO6b. Creating together joint values SO6c. Sharing love with others

### Step 3. Theory-Based Method and Practical Strategies for Program Design

Based on the results from steps 1 and 2, we developed an online-coaching blended couple-oriented intervention. We selected self-regulated learning (SRL) based on social cognitive theory for theoretical teaching methods and practical strategies to change the determinants of each level because it is widely used in blended programs (24, 25).

We developed a blended intervention that incorporated both online programs and coaching *via* videoconference. An online-coaching blended couple-oriented intervention provides opportunities to apply effective methods in an innovative manner. It also allows couples to manage and control their learning activities at their pace and receive real-time mental health care as per their convenience and time. In this blended intervention, we used methods of organization, rehearsal, elaboration, record keeping, and monitoring to improve knowledge, attitude, and skills at the intrapersonal level. In addition, reflective dialogue and performance feedback were applied to increase relationship functioning and shared outcomes at interpersonal and temporal/transpersonal levels, respectively.

These methods have been translated into practical strategies. The organization method was utilized to structure video lectures, such as learning objectives, case studies, and summaries of the main points. For rehearsal, we provided audio and video materials on mobile and web-based platforms to watch the videos repeatedly. We applied the method of elaboration to connect new things and prior knowledge by answering guided questions such as, “Describe your own situation relevant to the learning contents.”

Record keeping and monitoring were used to monitor their thinking and behavior to modify their behaviors. We encouraged couples to apply what they had learned in their lives and to write about their thoughts, emotions, and experiences on the mobile and web-based platforms. In conjoint sessions, we encouraged couples to share their writing with their spouses and complete tasks together.

For reflective dialogue and performance feedback, the coach started the sessions with quick reminders of the objectives and activities of the sessions to reflect on what they learned, and which of the activities helped them. In addition, the coach provided tailored feedback by analyzing their written responses to tasks.

### Step 4. Program Development

**Figure 1:**
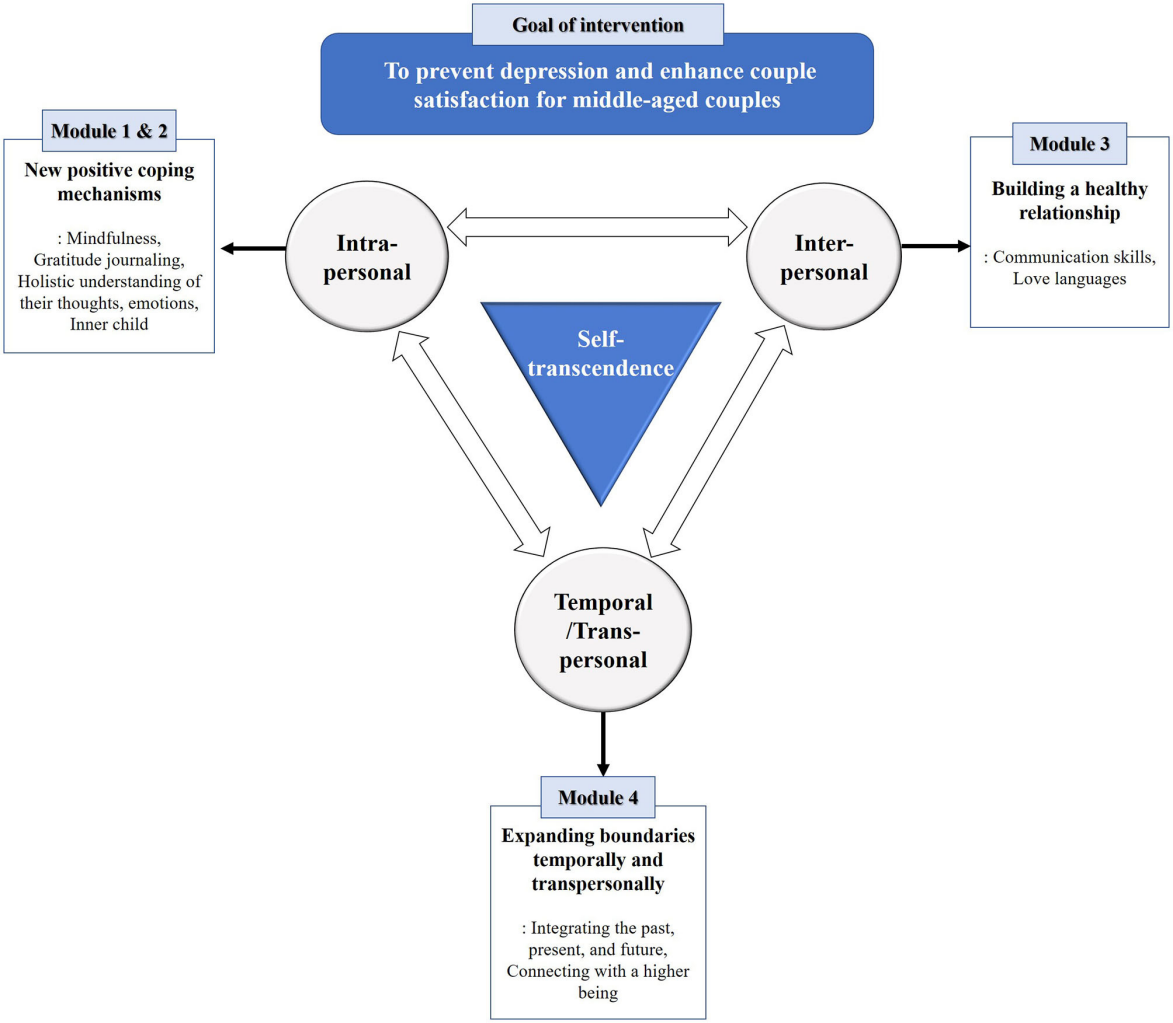
Theoretical framework of the self-transcendence theory.

The self-transcendence theory was selected as the theoretical framework to develop the *MindGuide Couple* program (Figure 1), which was designed to expand one's personal boundaries multidimensionally (i.e., at the intrapersonal, interpersonal, temporal, and transpersonal levels) to achieve POs.

For the enlargement of intrapersonal boundaries, Modules 1 and 2 were composed of new positive coping mechanisms, such as mindfulness (26) and gratitude journaling (27), to strengthen inner resources and a holistic understanding of their thoughts, emotions, and inner child to increase self-awareness. To extend the interpersonal boundaries, Module 3 focused on building a healthy relationship with the spouse by training effective couples' communication skills and committing to practicing the spouse's love languages. Module 4 was designed to expand boundaries at the temporal and transpersonal levels by integrating the past, present, and future and to connect with a higher being.

Modules 1 and 2 were designed as individual sessions, in which each spouse worked separately. Modules 3 and 4 were planned as conjoint sessions, wherein couples completed structured interaction tasks. The four modules consisted of 16 sessions that provided integrative techniques from different therapies to accomplish POs (Table 2). Each session took about 20–30 min to complete, depending on the individual learning abilities. Videoconference calls with a professional coach were planned to support couples at the end of each module. Coaches helped couples apply program content, reinforce relationships, and improve communication for 1 h.

**Table 2:** The contents of the *MindGuide Couple* program.

				**Blended contents**		
**Theme of module**	**PO**	**CO**	**Session**	**Online lectures**	**Tasks**	**Coaching**
Intrapersonal	PO1 PO2 PO3	A1a	1	Introduction to the program		Making couples set their goals Focusing on the present and future Helping couples perceive where they currently stand in their life
		K2a, A2a, S2a	2	Learn the mindfulness and gratitude journal	Mindfulness Writing a gratitude journal	
		K1a, K1b, K1c, A1b, R1a	3	Learn how to prevent depression and suicide	Mindfulness Depression screening test Writing a gratitude journal	
		A3a	4	Life map	Mindfulness Making a life map	
Intrapersonal	PO2 PO3 PO4	K2a, A4a	5	Discovering my true self	Mindfulness	Motivating couples with the required encouragement and support Helping couples understand how their minds operate differently Encouraging couples to be more self-aware Supporting to become a better person for their spouse Trying to ensure the couple's progress
		K3a, S3a	6	Exploring my inner self	Mindfulness Emotional focusing To find thinking-feeling connection	
		K3a, A2a, S2a, S3a	7	Inner child	Mindfulness Discovering my inner child Healing dialog	
Interpersonal	PO4 PO5	K4a, K4b, K4c	8	Communication style	Mindfulness Communication style test Understanding the characteristics of different communication style	Accepting yourself and your spouse's personality and characteristics differences Addressing communication problems between a couple Emphasizing self-responsibility rather than blaming the spouse Supporting during couples' communication Identifying a better way for couple relationship Helping the couple understand each other better Making couples act towards what they want for a better relationship Focusing on motivating couples toward a positive transition Restoring the couple relationship back to a balanced one
		K4a, K4b, K4c, A4a	9	Discover negative patterns of communication	Mindfulness Writing about negative patterns of communication each other	
		A4a, S4a, R4a	10	Identifying a conflict	Mindfulness Writing a conflict situation between a couple	
		A4a, S4a, R4a, R4b, SO4a	11	Exercises communication skills (1)	Mindfulness Exercises to develop communication skills (2)	
		K5a, A5b, S5a, R5a, SO4a	12	Exercises communication skills (2)	Mindfulness Exercises to develop communication skills (2)	
		K5a, A5a A5b, SO5a	13	Discovering your love language	5 love languages test Practical ways to speak the spouse's love language	
Interpersonal [Temporal and transpersonal	PO6	K6a, A6a, R6a	14	To find values	Mindfulness How to find values How to find meaning of life	Communicating in a way that leads to deep understanding and shared meaning Helping couples obtain relational oneness Focusing on planning the couple's future Encouraging couples walk together toward their Creator-given dreams and desires
		A6a, S6a, R6a, S6a, SO6b	15	Building a healthy relationship	Mindfulness Creating a balanced relationship Creating vision for couple	
		R6a, SO6a, SO6b	16	Finding happiness as a couple	Mindfulness The blessing of love Writing love letter to spouse The pledge of love	

**Figure 2:**
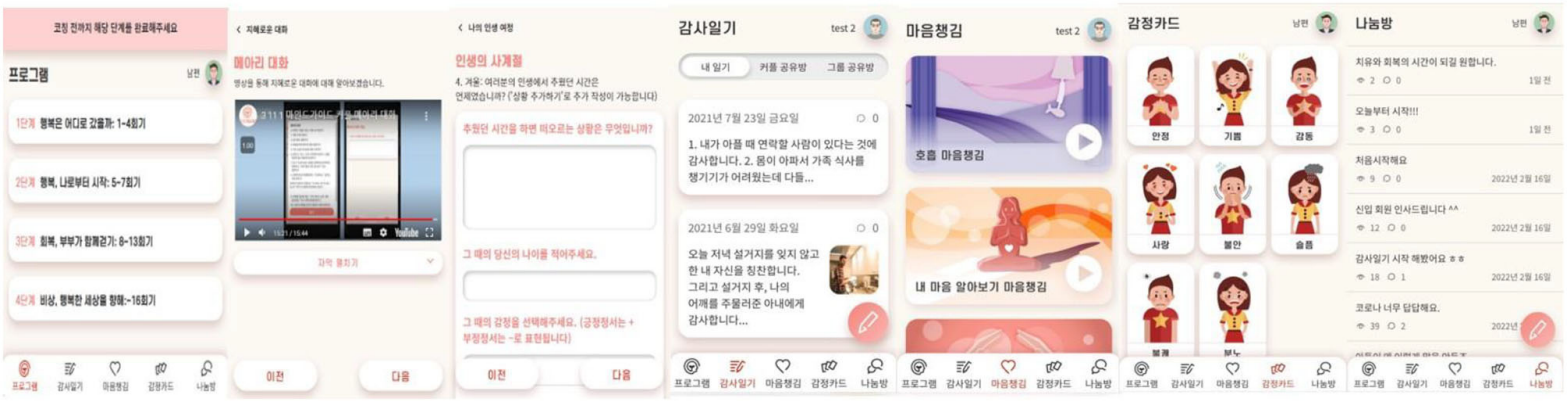
Screenshots of the *MindGuide Couple* program.

The *MindGuide Couple* program was developed through collaboration with interdisciplinary teams of one researcher, two psychiatric mental health nurse practitioners, one marriage and family therapist, one web designer, and two technical programmers. It was delivered online *via* mobile and web-based platforms and was divided into two pages: a user service page and an administrator dashboard. The user service pages contained (a) the *MindGuide Couple* program; (b) a gratitude journal; (c) mindfulness; (d) emotion cards, and (e) a discussion board. On the page of the *MindGuide Couple* program, participants engaged in the program and viewed their spouses' progress. In gratitude journal pages, participants wrote about one thing they were grateful for in a day. This journal was intended to help participants focus on emotional experiences. Participants could choose whether to disclose their journals to their spouses. Those desiring privacy could restrict access to the journals themselves. The administrator dashboard provides convenient management tools for the research team to organize and manage the web usage data. The *MindGuide Couple* program was developed and provided *via* mobile and web-based platforms (Figure 2).

#### Content Validation

Two couple and family therapists (CFTs), and three psychiatric mental-health nurse practitioners (PMHNPs) with practical experience in couple counseling and therapy evaluated the content validity of the program. The panel group was different from the experts in step 1. The content validity index (CVI) of a program is defined as the extent to which program components are relevant to the program goal and effectiveness in a target population (28). We developed 10 items of the assessment instrument for the CVI based on previous studies regarding the blended learning approach, program components and sequence, techniques, session length and frequency, and program outcomes (i.e., mental health and couple relationship) (28, 29). Five experts evaluated the CVI using a four-point Likert Scale ranging from 1 (not valid at all) to 4 (very valid). The CVI result was 1.0, indicating that the program had acceptable CVI values (30). Each member of the panel took an overall look at the program individually and independently, evaluated the content validation, and provided feedback. Moreover, the panel group held a videoconference meeting to generate a consensus between different opinions.

#### Coaching Training

Three coaches, one couple, a family therapist, and two mental health nurses, were trained in the coaching sessions. They hold either a doctoral or master's degree and have experience as couples and family therapists. Coaches received training on a series of couple therapies and experienced the program once per week for 2 months. The coaching protocol provided the program information and outlined the structure of the coaching process using a semi-structured script. Coaches received regular coaching supervision from the first author.

### Steps 5 and 6. Designing a Program Implementation and Evaluation Plan

The intervention implementation plan focused on the inclusion criteria for the participants and a plan for recruitment. The inclusion criteria for participation in the intervention will be as follows: (a) heterosexually married; (b) between the ages of 40 and 65 years; and (c) agreement to participate in the program. The exclusion criteria will be as follows: (a) having a spouse who was diagnosed with depression or other mental diseases; and (b) currently attending couple therapy and counseling and receiving drug treatment. When people are enrolled *via* the website, the research assistant will contact them for eligibility screening.

Participants will be recruited through advertisements, including study information on the *MindGuide Couple* website and several online communities, churches, and organizations (e.g., mental health and welfare centers, family support and treatment centers, and volunteer centers).

The effectiveness of the developed intervention will be evaluated in two stages. In the first stage, a feasibility study will aim to evaluate the feasibility, acceptability, usability, and preliminary efficacy of the intervention. In the second stage, we will apply the intervention to at-risk middle-aged couples and evaluate the effectiveness of the intervention on depressive symptoms and couple relationships. The preliminary effects of the program will be examined with primary outcomes, including the Center for Epidemiological Studies Depression Scale (31), Patient Health Questionnaire-9 (32), Generalized Anxiety Disorder 7-item (33), and Positive and Negative Affect Schedule (34). Secondary outcomes using the Couple Satisfaction Index 16-item (35), Family Relationship Assessment Scale (36), and the Satisfaction with Life Scale (37) will be evaluated before the program, immediately after program completion, and 2 months following the program.

## Discussion

We described the development, implementation, and evaluation of an online-coaching blended couple-oriented intervention using an online program and coaching, called the *MindGuide Couple* program using IM. The IM approach appears useful in developing this tailored intervention that is specific to middle-aged couples' mental health. As aging enhances emotional sensitivity to stress, middle-aged adults are vulnerable to depression (3, 38). Furthermore, depression in mid-life increases the risk of dementia in late-life (39). Thus, preventing mid-life depressive symptoms is essential for improving the quality of life in older adults. In particular, because relationship distress in couples is associated with a higher risk of depression (8), couple-oriented interventions may provide the opportunity for middle-aged couples to prevent depression and promote couple relationships as well.

The *MindGuide Couple* program was developed by conducting a scoping review, a quantitative Borich's Needs Assessment of middle-aged couples, and an Integrative Needs Assessment process, including qualitative couple interviews with regard to couple-oriented intervention and expert interviews. Fernandez et al. (40) highlighted the need for the identification of multiple target adopters and implementers to develop a multi-level intervention. Consistent with this suggestion, we used couple interviews (41, 42) to collect data at both the individual and couple level. In addition, interpretative phenomenological analysis (42) was used to assess both the individuals' and couples' needs by exploring not only personal experiences but also shared couples' experiences of psychological problems in the context of interdependent relationships.

Another strength of this program is that it is structured around the self-transcendence theory, which is suitable for promoting middle-aged couples' psychological wellbeing (43, 44). The program helps participants gradually expand self-boundaries in multidimensional ways, according to the program modules. For example, in Modules 1 and 2 of the program, participants learned intrapersonal skills, such as self-awareness, mindfulness, and gratitude journaling. To expand interpersonal boundaries by building a healthy relationship with the spouse, Module 3 provides opportunities for understanding their spouse's emotions and needs, accepting the spouse's inner child, and helping the spouse heal their inner child. Finally, in Module 4, they develop a couple's mission, vision, and value statements together by integrating them with their past, present, and future.

The distinctive strength of this online-coaching blended program is that it involves both flexible schedule online learning on mobile and web-based platforms, and vis-a-vis coaching using videoconference. Several researchers have suggested that online programs may provide opportunities for couples to overcome mental health stigma, lack of financial resources, and geographic barriers and to increase access to the program (45, 46). In addition, considering the busy lives of middle-aged couples, this online-coaching blended *MindGuide Couple* program allows them to participate in the program from any place when they have time and energy. Moreover, this blended couple-oriented intervention with coaching can enhance and expand couple-oriented interventions to facilitate real-time interaction between a couple and coach (45).

In this study, the program was developed with a target population of middle-aged couples, marriage, and family therapists, and a literature review was conducted. However, further research is needed to evaluate the feasibility, acceptability, and preliminary effects of the *MindGuide Couple* program on mental health (e.g., depression and anxiety) and couple relationships (e.g., couple satisfaction). Another limitation of this study is that the program was developed at both the individual and couple levels; however, family and community levels also need to be considered when developing multi-level interventions.

## Conclusion

This article provides a detailed description of how we systematically developed an online-coaching blended couple-oriented intervention for preventing depression and promoting couple relationships based on IM. The IM allowed us to develop a tailored multi-level intervention based on the needs of middle-aged couples. This newly developed online-coaching blended couple-oriented intervention may provide opportunities for couples to overcome mental health stigma and enhance real-time interaction between a couple and a coach. The primary effects of this newly developed program should be evaluated in future studies.

## Data Availability Statement

The raw data supporting the conclusions of this article will be made available by the authors, without undue reservation.

## Ethics Statement

The studies involving human participants were reviewed and approved by Institutional Review Board of Ewha Womans University. The participants provided their written informed consent to participate in this study.

## Author Contributions

S-SK, MG, and DK: study and manuscript conceptualization. S-SK and MG: contributed to methods, and results. S-SK and DK: contributed to background and results. S-SK: contributed to discussion. All authors contributed to the article and approved the submitted version.

## Funding

This work was supported by the National Research Foundation of Korea (NRF) grant funded by the Korea government (MSIT) (No. 2019R1H1A2039669, 2019R1A2C1087398, and 2022R1A2C2004867).

## Conflict of Interest

The authors declare that the research was conducted in the absence of any commercial or financial relationships that could be construed as a potential conflict of interest.

## Publisher's Note

All claims expressed in this article are solely those of the authors and do not necessarily represent those of their affiliated organizations, or those of the publisher, the editors and the reviewers. Any product that may be evaluated in this article, or claim that may be made by its manufacturer, is not guaranteed or endorsed by the publisher.
